# Consumption of a high-fat diet alters transcriptional rhythmicity in liver from pubertal mice

**DOI:** 10.3389/fnut.2022.1068350

**Published:** 2023-01-04

**Authors:** Lin Yan, Sneha Sundaram, Bret M. Rust, Daniel G. Palmer, LuAnn K. Johnson, Huawei Zeng

**Affiliations:** United States Department of Agriculture, Agricultural Research Service, Grand Forks Human Nutrition Research Center, Grand Forks, ND, United States

**Keywords:** circadian rhythms, metabolism, diet, puberty, mice

## Abstract

**Introduction:**

Childhood obesity is associated with adult obesity, which is a risk factor for chronic diseases. Obesity, as an environmental cue, alters circadian rhythms. The hypothesis of this study was that consumption of a high-fat diet alters metabolic rhythms in pubertal mice.

**Methods:**

Weanling female C57BL/6NHsd mice were fed a standard AIN93G diet or a high-fat diet (HFD) for 3 weeks. Livers were collected from six-week-old mice every 4 h over a period of 48 h for transcriptome analysis.

**Results and discussion:**

The HFD altered rhythmicity of differentially rhythmic transcripts in liver. Specifically, the HFD elevated expression of circadian genes *Clock*, *Per1*, and *Cry1* and genes encoding lipid metabolism *Fads1* and *Fads2*, while decreased expression of circadian genes *Bmal1* and *Per2* and lipid metabolism genes *Acaca*, *Fasn*, and *Scd1*. Hierarchical clustering analysis of differential expression genes showed that the HFD-mediated metabolic disturbance was most active in the dark phase, ranging from Zeitgeber time 16 to 20. The Kyoto Encyclopedia of Genes and Genomes enrichment analysis of differentially expressed genes showed that the HFD up-regulated signaling pathways related to fatty acid and lipid metabolism, steroid and steroid hormone biosynthesis, amino acid metabolism and protein processing in the endoplasmic reticulum, glutathione metabolism, and ascorbate and aldarate metabolism in the dark phase. Down-regulations included MAPK pathway, lipolysis in adipocytes, Ras and Rap1 pathways, and pathways related to focal adhesion, cell adhesion molecules, and extracellular matrix-receptor interaction. In summary, the HFD altered metabolic rhythms in pubertal mice with the greatest alterations in the dark phase. These alterations may disrupt metabolic homeostasis in puberty and lead to metabolic disorders.

## Introduction

Drastic increases in childhood obesity since the last century have created a critical public health challenge for the 21 century (1, 2). Childhood obesity is associated with adult obesity (3), which is related to chronic diseases. These include coronary heart disease (4, 5), diabetes (5–7), and certain cancer (8).

Obesity is a risk factor for metabolic disorders. Obese patients often have symptoms of metabolic syndrome, which is clinically defined as having three of five conditions: excessive body fat, abnormal cholesterol or triacylglycerol levels, high blood sugar, and high blood pressure. These symptoms occur in rodent models of obesity with disturbed diurnal rhythms (9, 10).

All mammals exhibit diurnal rhythms, cycling for approximately every 24 h, emerging from interactions between internal circadian clocks and the natural light/dark cycle (11–13). A consistent daily living pattern (e.g., eating vs. fasting, sleep vs. awake) maintains the normal rhythms, whereas frequent disruptions to the existing pattern alter the daily rhythms of oscillations. Obesity is an environmental cue that disrupts circadian rhythms and results in metabolic dysfunction (14–16).

All biological processes, ranging from metabolic pathways to physiological functions, are driven by the central clock and peripheral oscillators. The central clock at the suprachiasmatic nucleus in hypothalamus controls diurnal rhythms by generating downstream signals synchronized to the light/dark cycles. These signals synchronize peripheral oscillators, which are autonomous circadian oscillators present in cells of all organs. The peripheral clocks respond to environmental alterations (e.g., diet) but rely upon the central clock for synchronization. The synchrony between the central and peripheral clocks sets a temporal rhythmic control of homeostatic regulation for health and wellbeing (11–13).

The molecular machinery that generates circadian rhythms is an interconnected transcription-translation feedback loop that regulates diurnal patterns of circadian gene expression. Formation of circadian locomotor output cycles kaput/aryl hydrocarbon receptor nuclear translocator-like protein 1 (*Clock/Bmal1*) heterodimers in nucleus during the rest phase induces transcription of period (*Per*) and cryptochrome (*Cry*). Period and cryptochrome proteins accumulate in cytoplasm at the late rest phase or early active phase and form dimers that translocate back to nucleus to repress *Clock/Bmal1* transcription. This process cycles approximately every 24 h. In addition, the *Clock/Bmal1* heterodimers activate transcription of nuclear receptor subfamily 1 group D member 1 (*Rev-erb*), which represses the expression of *Clock/Bmal1*.

Metabolic dysfunction occurs in laboratory rodents fed an obesogenic diet (10, 17). Mice fed a high-fat diet consume more diet in the light phase than in the dark phase (14). This alteration leads to phase-advance of *Per2* in liver (15) and blunts the rhythmic expression of *Clock*, *Bmal1*, and *Per2* in liver (16, 18) and adipose tissue (16). The altered expression of circadian genes occurs in diet-induced obese mice with increased body adiposity and elevated tissue inflammation (14).

Puberty is a period of rapid development and growth; diet quality affects the progression of puberty. Dietary intervention by feeding mice a high-fat diet from weanling to adulthood increases body fat mass (10, 19), disturbs the daily oscillations of energy expenditure (9, 10), and alters rhythmic expression of circadian genes (14–16). However, the impact of dietary fat on metabolic homeostasis in pubertal mice remains to be elucidated. We hypothesized that consumption of a high-fat diet alters metabolic rhythms in pubertal mice. This study investigated the effect of a three-week feeding of a high-fat diet on metabolism in pubertal mice. It focused on changes in diurnal expression of circadian genes, genes encoding lipid metabolism, and metabolic signaling pathways identified by the analysis of differentially expressed genes.

## Materials and methods

### Animals and diets

Mice (C57BL/6NHsd, female, three-week-old) were purchased from Envigo, Madison, WI, USA. They were housed in a pathogen-free room with a 12:12-hour light/dark cycle (light intensity was 32 lux during the light phase). The room temperature was 22 ± 1°C. Mice were group-housed to avoid single-housing related stress. The AIN93G standard diet (20) and a high-fat diet (HFD, a modified AIN93G formulation) were used in this study, in which soybean oil is the source of dietary fat (Table 1). Soybean oil contains 14.9% saturated fatty acids, 22.1% monounsaturated fatty acids, and 57.6% polyunsaturated fatty acids.^1^ Diets were kept at −20°C and provided to mice every other day.

**TABLE 1:** Composition of diets.

Ingredient	AIN93G	High-fat
	**g/kg**	**g/kg**
Corn starch	397.5	42.5
Casein	200	239.4
Dextrin	132	239.4
Sucrose	100	119.7
Soybean oil	70	239.4
Cellulose	50	59.8
AIN93 mineral mix	35	41.9
AIN93 vitamin mix	10	12
L-Cystine	3	3.6
Choline bitartrate	2.5	3
*t*-Butylhydroquinone	0.014	0.017
Total	1,000	1,000
Energy	%	%
Protein	20	20
Fat	16	45
Carbohydrate	64	35
Analyzed gross energy^a^ kcal/g	4.3 ± 0.1	5.2 ± 0.1

^a^Quantified by oxygen bomb calorimeter (Model 6200, Parr Instrument, Moline, IL, USA). Values are means ± SEM (*n* = 3 samples per diet).

### Experimental design

Mice were acclimated with the AIN93G diet for 2 days before they were randomly assigned to two groups of 70 each. Mice had free access to their diets (AIN93G or HFD) and deionized water for 3 weeks; they were weighed weekly. At termination, five mice from each group were intraperitoneally injected with a mixture of ketamine/xylazine for euthanasia followed by exsanguination at Zeitgeber time (ZT) 0, 4, 8, 12, 16, 20, and 24 over a period of 48 h (light on at ZT 0 and off at ZT 12). Their livers were collected and stored at −80°C. Mice were not fasted prior to termination to prevent disturbing circadian rhythms.

### Real-time quantitative PCR

Total RNA from frozen liver was isolated by using the RNeasy Lipid Tissue Mini Kit (Qiagen, Germantown, MD, USA). The purity of the isolated RNA was analyzed by using the NanoDrop 8000 Spectrophotometer (Thermo Scientific, Wilmington, DE, USA). Complementary DNA (cDNA) was synthesized by using the high-capacity cDNA reverse transcription kit (Applied Biosystems, Waltham, MA, USA). Real-time quantitative PCR of circadian locomotor output cycles kaput (*Clock*) (Mm00455950_ml), aryl hydrocarbon receptor nuclear translocator-like protein 1 (*Bmal1*/*Arntl*) (Mm00500223_ml), period-1 (*Per1*) and *Per2* (Mm00501813_ml and Mm00478099_ml), cryptochrome 1 (*Cry1*) (Mm00514392_ml), nuclear receptor subfamily 1 group D member 1 (*Nr1d1/1Rev-erbα*) (Mm00520708_ml), acetyl-CoA carboxylase (*Acaca*) (Mm01304257_m1), fatty acid desaturase (*Fads*) *1* and *2* (Mm00507605_m1 and Mm00517221_m1), fatty acid synthase (*Fasn*) (Mm00662319_m1), stearoyl-CoA desaturase 1 (*Scd1*) (Mm00772290_m1), and sterol regulatory element-binding protein 1 (*Srebf1*) (Mm00550338_m1) was analyzed and normalized to the 18s rRNA by using the TaqMan Assay of Demand primers on the ABI QuantStudio 12K-Flex Real-time PCR system (Applied Biosystems). Probes for aforementioned genes and 18s rRNA were purchased from Applied Biosystems. Changes in gene expression were calculated by using the 2^–ΔΔCT^ method (21). The protein-protein interaction network analysis based on transcriptional data for circadian genes and genes encoding lipid metabolism was performed by using the STRING database^2^ (22, 23).

### RNA sequencing analysis

Total RNA extractions (three from each time point per group, ZT 0 to ZT 24) described above were used for RNA sequencing analysis. RNA quality was examined by using the Fragment Analyzer with the RNA analysis Kit DNF-471-500 (Advanced Analytical Technologies, Ames, IA, USA). Samples with an RNA Quality Number ≥ 7.3 were used for library construction, sequencing, and bioinformatics analysis (Novogene, Sacramento, CA, USA). Genes with read counts of 25 or greater were retained for the analysis (24, 25). Differential rhythmicity of transcripts between the two groups were compared by using R package compareRhythms (26). The default settings model_selection argument was used with the change of just_classify argument to FALSE so that to use the same set of data for the bar chart and graph in circular plot. The hierarchical clustering analysis of differentially expressed genes was performed by using R package pheatmap (27) (Novogene). The Kyoto Encyclopedia of Genes and Genomes (KEGG) enrichment analysis of the differentially expressed genes was performed by using R package clusterProfiler for comparing biological themes among gene clusters (28, 29) (Novogene).

### Quantification of hepatic cholesterol and triacylglycerols

Liver samples (*n* = 14, two from each time point per group) were prepared by using the method described previously (30). Total cholesterol and triacylglycerols were quantified by using the COBAS Integra 400 Plus Analyzer (Roche Diagnostics, Indianapolis, IN, USA) with corresponding test kits #03039773 and #20767107 (Roche Diagnostics). Data were normalized by liver weight before statistical analysis.

### Statistical analyses

Student *t*-test was performed for comparisons between the AIN93G and HFD groups (means ± standard error of the mean, SEM). The Cosinor model was performed for differences in rhythmic expression of circadian and lipid metabolism genes in liver between the two groups. The model y = Mesor + amplitude × Cos [2π/24 × (*t*–acrophase)]. Mesor (midline estimating statistic of rhythm) is the mean of the oscillations, amplitude is the distance between Mesor and the peak of oscillations, acrophase is the hour at which the maximum value of the cosine wave (the peak of the rhythm) occurs, and *t* is time in hours. The period of oscillations was assumed to be 24 h. Nested non-linear models were used to determine if separate model parameters (Mesor, amplitude, and acrophase) were needed for each group or if common parameters were sufficient. In these analyses, parameters were estimated directly for the AIN93G group, while the parameters for the HFD group were expressed as offsets from the corresponding AIN93G parameters. If the 95% confidence interval (95% CI) of the offset did not cross 0, the parameters were considered different between the AIN93G and HFD groups (*p* ≤ 0.05) (31). All models were fit by using the NLIN procedure in SAS 9.4 (SAS Institute, Inc., Cary, NC, USA). Differential rhythmicity of transcripts between the two groups were compared by using R (24–29, 32).

## Results

### Body weight

Mice fed the HFD were slightly but significantly heavier than mice fed the AIN93G diet. Initial body weight was 10.4 ± 0.1 g (*n* = 140) at randomization. Body weight was 19.0 ± 0.2 g and 18.2 ± 0.2 (*p* < 0.05, *n* = 70 per group) for the HFD-fed and AIN93G-fed mice, respectively, at the end of the study.

### Hepatic content of cholesterol and triacylglycerols

There was no difference in total cholesterol between HFD-fed and AIN93G-fed mice (1.84 ± 0.10 vs. 1.64 ± 0.09 mg/g, *p* = 0.17, *n* = 14 per group). The content of triacylglycerols was 66% greater in HFD-fed mice than in their AIN93G-fed counterparts (23.60 ± 1.59 vs. 14.22 ± 0.68 mg/g, *p* < 0.01, *n* = 14 per group).

### Hepatic expression of circadian genes

**FIGURE 1:**
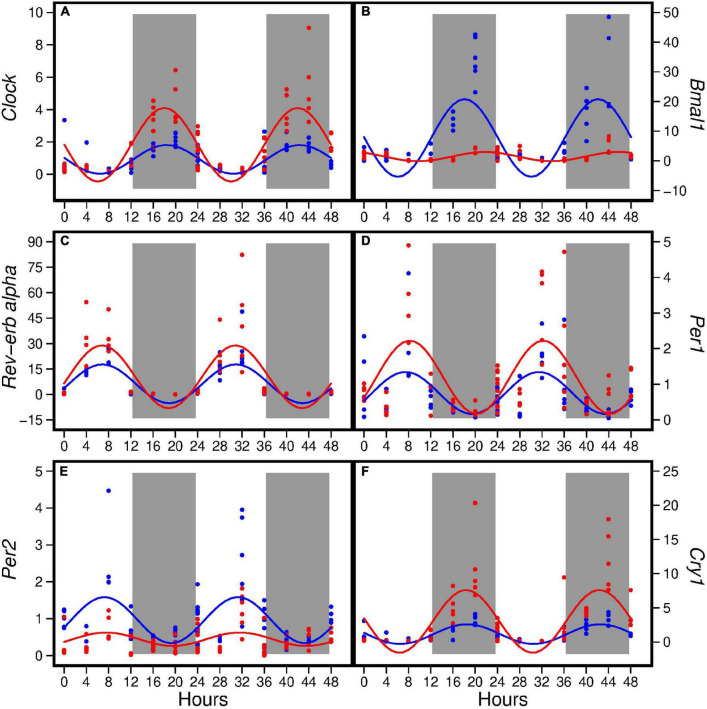
Hepatic expression of *Clock* **(A)**, *Bmal1* **(B)**, *Rev-erbα* **(C)**, *Per1* **(D)**, *Per2* **(E)**, and *Cry1* **(F)** in pubertal mice fed the AIN93G or high-fat diet (*n* = 5 per time point per group). Values from mice terminated in the light phase are fold changes to the AIN93G group at ZT 0 and that from mice terminated in the dark phase are fold changes to AIN93G group at ZT 12. Open background: light phase; gray background: dark phase; blue: AIN93G diet; red: high-fat diet. The rhythm curves were generated by using the Cosinor model y = Mesor + amplitude × Cos [2π/24 × (*t*–acrophase)].

The HFD disturbed rhythmic expression of circadian genes in liver (Figure 1). Of the six circadian genes analyzed, the HFD elevated amplitude of *Clock, Rev-erbα*, *Per1*, and *Cry1* (ranging from 62 to 219%) and decreased amplitude of *Bmal1* and *Per2* by 88 and 71%, respectively, (Table 2). Similarly, the HFD elevated Mesor of *Clock, Rev-erbα*, *Per1*, and *Cry1* (ranging from 58 to 163%) and decreased Mesor of *Bmal1* and *Per2* by 81 and 53%, respectively, (Table 2). The acrophases of these genes did not differ between the two groups (Table 2). When data from all seven time points were combined, the HFD increased the expression of *Clock*, *Per1*, and *Cry1* and decreased that of *Bmal1* and *Per2* when compared to the AIN93G diet (Table 3).

**TABLE 2:** Fold changes in estimated statistic of rhythm in the expression of circadian genes in liver from pubertal mice fed the AIN93G or high-fat diet.

mRNA	AIN93G^a^	High-fat^a^	Difference (95% CI)^b^	*p*
**Amplitude**				
*Clock*	0.89 ± 0.17	2.27 ± 0.17	1.38 (0.91–1.85)	<0.01
*Bmal1*	13.09 ± 1.16	1.55 ± 1.06	−11.54 (−14.64–−8.43)	<0.01
*Rev-erbα*	11.44 ± 1.69	18.53 ± 1.67	7.08 (2.39–11.78)	<0.01
*Per1*	0.59 ± 0.15	1.03 ± 0.14	0.44 (0.03–0.85)	<0.05
*Per2*	0.62 ± 0.11	0.18 ± 0.11	−0.44 (−0.75–−0.14)	<0.01
*Cry1*	1.44 ± 0.42	4.59 ± 0.42	3.15 (1.98–4.32)	<0.01
**Acrophase**				
*Clock*	18.38 ± 0.64	17.99 ± 0.25	−0.38 (−1.74–0.98)	0.58
*Bmal1*	18.08 ± 0.30	21.76 ± 2.70	3.68 (−1.70–9.06)	0.18
*Rev-erbα*	6.88 ± 0.50	6.80 ± 0.31	−0.08 (−1.24–1.09)	0.90
*Per1*	7.45 ± 0.88	8.23 ± 0.51	0.78 (−1.22–2.78)	0.44
*Per2*	7.25 ± 0.60	7.55 ± 2.06	0.30 (−3.95–4.54)	0.89
*Cry1*	18.50 ± 0.99	18.37 ± 0.31	−0.14 (−2.18–1.91)	0.90
**Midline estimating statistic of rhythm**				
*Clock*	0.92 ± 0.11	1.83 ± 0.11	0.91 (0.59–1.22)	<0.01
*Bmal1*	7.71 ± 0.77	1.44 ± 0.76	−6.27 (−8.42–−4.13)	<0.01
*Rev-erbα*	6.27 ± 1.13	10.38 ± 1.12	4.10 (0.96–7.25)	0.01
*Per1*	0.76 ± 0.10	1.20 ± 0.10	0.44 (0.16–0.72)	<0.01
*Per2*	0.96 ± 0.07	0.45 ± 0.07	−0.52 (−0.72–−0.31)	<0.01
*Cry1*	1.15 ± 0.28	3.02 ± 0.28	1.87 (1.09–2.65)	<0.01

^a^Estimate ± standard error (SE).

^b^Estimated difference and its 95% confidence interval between the AIN93G and high-fat diet groups (*n* = 5 from each time point per group).

**TABLE 3:** Hepatic expression of circadian genes and genes encoding lipid metabolism in pubertal mice fed the AIN93G or high-fat diet.

Gene	AIN93G	High-fat	*p*
*Clock*	1 ± 0.10	1.92 ± 0.22	<0.01
*Bmal1*	1 ± 0.14	0.42 ± 0.07	<0.01
*Rev-erbα*	1 ± 0.20	1.63 ± 0.34	0.11
*Per1*	1 ± 0.13	1.52 ± 0.20	0.04
*Per2*	1 ± 0.11	0.46 ± 0.05	<0.01
*Cry1*	1 ± 0.12	2.55 ± 0.41	<0.01
*Acaca*	1 ± 0.14	0.33 ± 0.05	<0.01
*Fads1*	1 ± 0.04	1.36 ± 0.05	<0.01
*Fads2*	1 ± 0.11	2.07 ± 0.15	<0.01
*Fasn*	1 ± 0.14	0.41 ± 0.06	<0.01
*Scd1*	1 ± 0.15	0.43 ± 0.07	<0.01
*Srebf1*	1 ± 0.08	0.83 ± 0.06	0.07

*N* = 70 per group.

### Hepatic expression of genes encoding lipid metabolism

**FIGURE 2:**
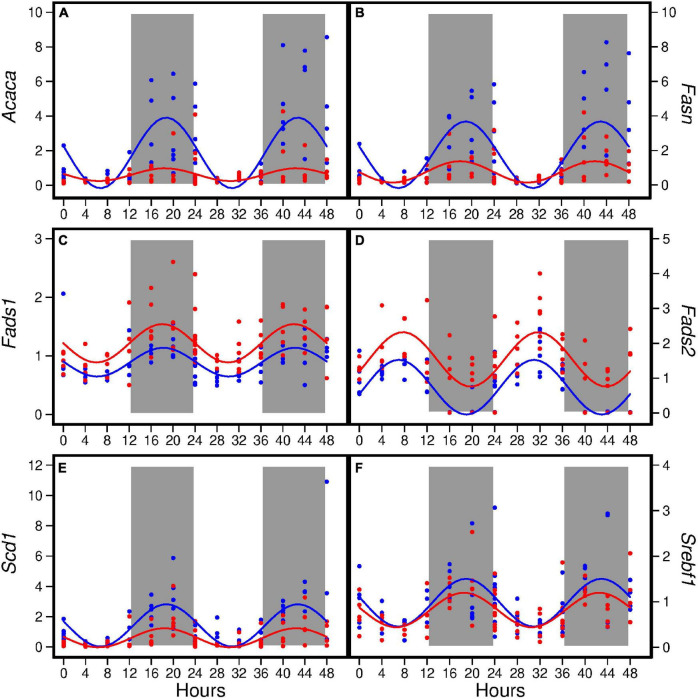
Hepatic expression of *Acaca* **(A)**, *Fasn* **(B)**, *Fads1* **(C)**, *Fads2* **(D)**, *Scd1* **(E)**, and *Srebf1* **(F)** in pubertal mice fed the AIN93G or high-fat diet (*n* = 5 per time point per group). Values from mice terminated in the light phase are fold changes to the AIN93G group at ZT 0 and that from mice terminated in the dark phase are fold changes to AIN93G group at ZT 12. Open background: light phase; gray background: dark phase; blue: AIN93G diet; red: high-fat diet. The rhythm curves were generated by using the Cosinor model y = Mesor + amplitude × Cos [2π/24 × (*t*–acrophase)].

The HFD altered diurnal expression of genes encoding lipid metabolism in liver (Figure 2). The HFD reduced amplitude of *Acaca, Fasn*, and *Scd1* (ranging from 55 to 82%) (Table 4). Similarly, the HFD reduced Mesor of *Acaca, Fasn*, and *Scd1* (ranging from 57 to 67%) but increased Mesor of *Fads1* and *Fads2* by 37 and 108%, respectively, (Table 4). The HFD did not affect acrophases of these genes (Table 4). In the combined analysis of data from all seven time points, the HFD decreased the expression of *Acaca*, *Fasn*, and *Scd1* and increased that of *Fads1* and *Fads2* when compared to the AIN93G diet (Table 3).

**TABLE 4:** Fold changes in estimated statistic of rhythm in the expression of genes encoding lipid metabolism in liver from pubertal mice fed the AIN93G or high-fat diet.

mRNA	AIN93G^a^	High-fat^a^	Difference (95% CI)^b^	*p*
**Amplitude**				
*Acaca*	2.04 ± 0.26	0.37 ± 0.26	−1.67 (−2.39–−0.95)	<0.01
*Fasn*	1.93 ± 0.24	0.62 ± 0.24	−1.31 (−1.98–−0.65)	<0.01
*Fads1*	0.25 ± 0.06	0.33 ± 0.06	0.08 (−0.08–0.24)	0.31
*Fads2*	0.78 ± 0.11	0.78 ± 0.11	0.01 (−0.30–0.30)	0.98
*Scd1*	1.40 ± 0.21	0.63 ± 0.21	−0.77 (−1.35–−0.18)	0.01
*Srebf1*	0.52 ± 0.09	0.40 ± 0.09	−0.13 (−0.38–0.12)	0.31
**Acrophase**				
*Acaca*	18.72 ± 0.43	18.44 ± 2.38	−0.28 (−5.05–4.50)	0.91
*Fasn*	18.95 ± 0.42	17.91 ± 1.30	−1.04 (−3.74–1.66)	0.45
*Fads1*	18.19 ± 0.79	18.03 ± 0.59	−0.16 (−2.12–1.89)	0.87
*Fads2*	6.98 ± 0.47	7.73 ± 0.48	0.75 (−0.58–2.08)	0.27
*Scd1*	18.73 ± 0.51	18.37 ± 1.11	−0.36 (−2.78–2.06)	0.77
*Srebf1*	18.91 ± 0.58	18.53 ± 0.76	−0.39 (−2.27–1.50)	0.69
**Midline estimating statistic of rhythm**				
*Acaca*	1.88 ± 0.17	0.62 ± 0.17	−1.26 (−1.74–−0.78)	<0.01
*Fasn*	1.75 ± 0.16	0.76 ± 0.16	−0.99 (−1.44–−0.55)	<0.01
*Fads1*	0.89 ± 0.04	1.22 ± 0.04	0.32 (0.21–0.43)	<0.01
*Fads2*	0.74 ± 0.07	1.54 ± 0.07	0.80 (0.60–1.00)	<0.01
*Scd1*	1.42 ± 0.14	0.61 ± 0.14	−0.82 (−1.21–−0.43)	<0.01
*Srebf1*	0.98 ± 0.06	0.82 ± 0.06	−0.16 (−0.33–0.01)	0.06

^a^Estimate ± standard error (SE).

^b^Estimated difference and its 95% confidence interval between the AIN93G and high-fat diet groups (*n* = 5 from each time point per group).

### Interaction analysis of circadian and lipid metabolism genes

**FIGURE 3:**
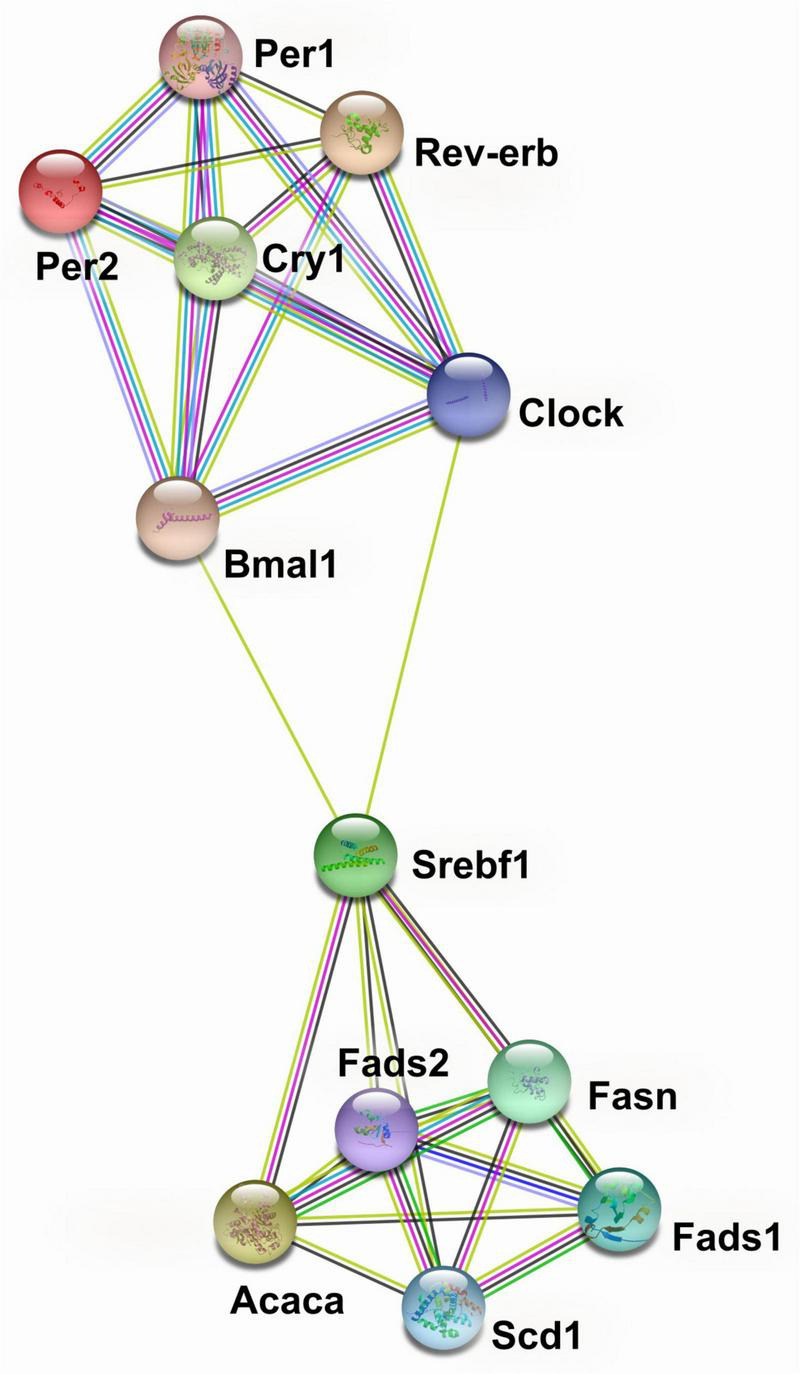
Protein-protein interaction network analysis of circadian genes and genes encoding lipid metabolism in liver from pubertal mice fed the AIN93G or high-fat diet. 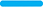 from curated databases; 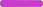 from expirermentally determined; 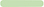 from text mining; 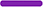 from protein homology; 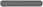 from co-expression; 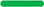 from gene neighborhood; 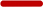 from gene fusions. The analysis was performed by using the STRING database (http://string-db.org).

The protein-protein interaction network analysis based on transcriptional data for circadian genes and genes encoding lipid metabolism showed that *Clock, Bmal1, Rev-erba, Per1, Per2*, and *Cry1* formed one cluster and that *Acaca, Fads1, Fads2, Fasn, Scd1*, and *Srebf1* formed another (Figure 3). The two clusters were bridged by *Clock* and *Bmal1* with *Srebf1* (Figure 3).

### Transcriptome analysis

**FIGURE 4:**
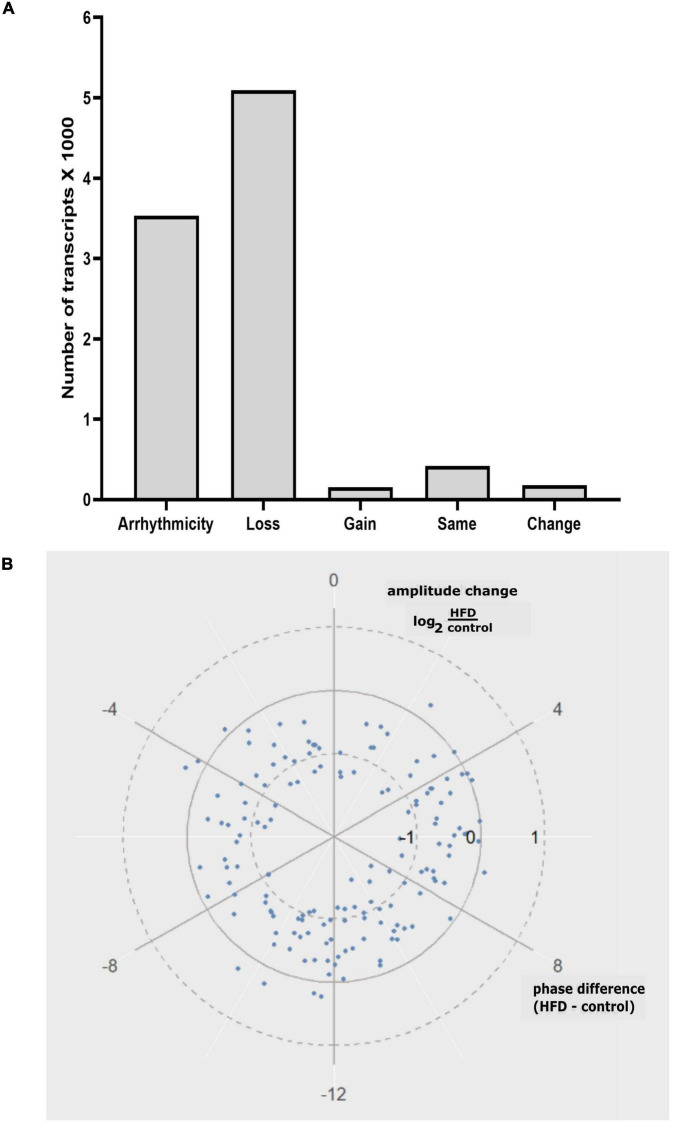
**(A)** Categorization of differentially rhythmic transcripts identified in this study: Arrhythmicity, Loss: loss of rhythms, Gain: gain of rhythms, Same: same rhythms, and Change: change of rhythms. **(B)** Circular plot representing the amplitude and phase changes in differentially rhythmic transcripts between the AIN93G and HFD groups.

The analysis of differentially rhythmic transcripts showed that there were 3,535 transcripts that were arrhythmic in this study (Figure 4A). There were 5,094 transcripts that lost rhythms, 156 gained rhythms, 181 showed changes in rhythmicity, and 421 remained the same rhythms without alterations between the two groups (Figure 4A). In the transcripts that showed changes, the circular plot showed that the rhythm amplitude of differentially rhythmic transcripts was largely reduced in the HFD group relative to the AIN93G group (Figure 4B). There was no clear trend in phase change (Figure 4B).

**FIGURE 5:**
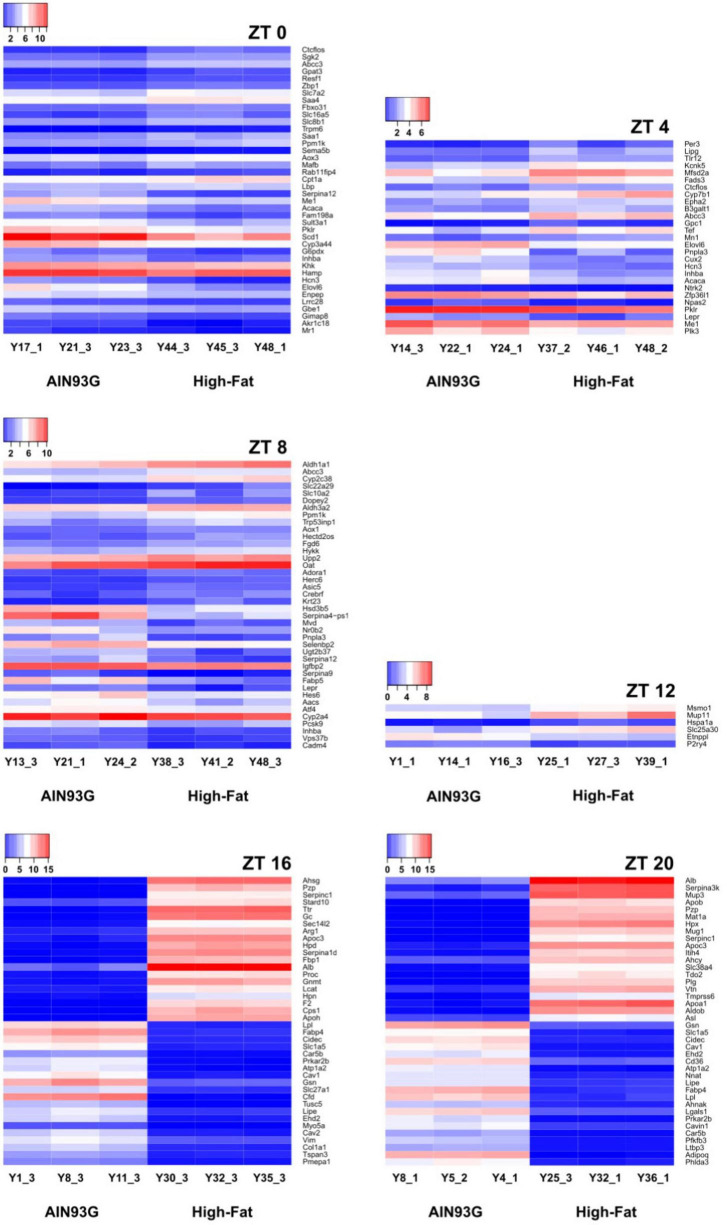
Hierarchical clustering heatmap of differentially expressed genes in liver from pubertal mice that are significantly altered by the high-fat diet. The 40 most differentially expressed genes are reported for ZT 0, 8, 16, and 20. Data not shown for ZT 24 (no significant differences in differentially expressed genes between the two groups). The color ranging from red to blue indicates that log2(FPKM+1) values are from large to small (*n* = 3 from each time point per group).

Hierarchical clustering analysis on differential expression compared genes with similar expression patterns between the AIN93G and HFD groups. The most differentially expressed genes between the two groups at each of the seven time points are presented in Figure 5. Compared to the AIN93G diet, the HFD had the greatest effect on differential expression at ZT 16 and ZT 20 of the dark phase (Figure 5).

**FIGURE 6:**
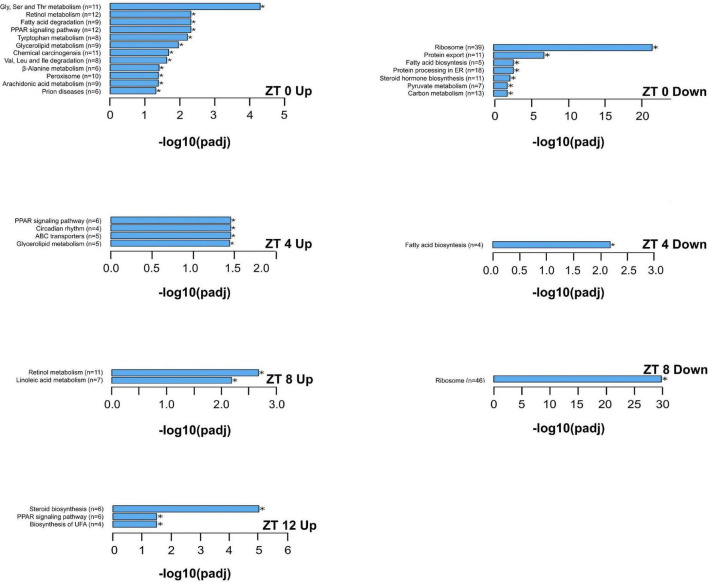
The upregulated and downregulated pathways and biological functions by the KEGG enrichment analysis of differentially expressed genes that are significantly altered by the high-fat diet in liver from pubertal mice for ZT 0, 4, 8, and 12 (adjusted *p* ≤ 0.05, *n* = 3 from each timepoint per group). Data not shown for downregulations at ZT 12 (no significant differences in KEGG terms between the two groups). Up: upregulated; Down: downregulated. Numbers in paratheses are numbers of differentially expressed genes identified. ABC transporters: ATP binding cassette transporters; ER: endoplasmic reticulum; PPAR: peroxisome proliferator-activated receptors; UFA: unsaturated fatty acids.

**FIGURE 7:**
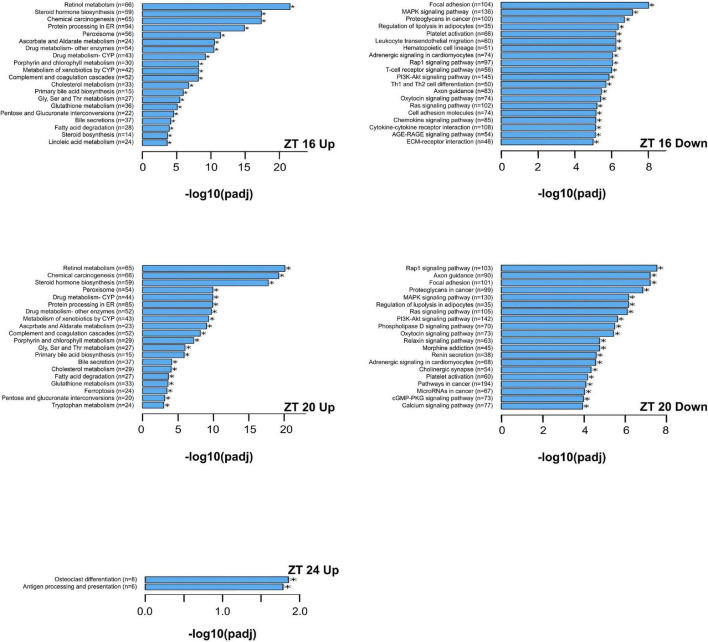
The upregulated and downregulated pathways and biological functions by the KEGG enrichment analysis of differentially expressed genes that are significantly altered by the high-fat diet in liver from pubertal mice for ZT 16, 20, and 24 (adjusted *p* ≤ 0.05, *n* = 3 from each timepoint per group). The 20 most upregulated and 20 most downregulated KEGG terms are reported for ZT 16 and 20. Data not shown for downregulations at ZT 24 (no significant differences in KEGG terms between the two groups). Up: upregulated; Down: downregulated. Numbers in paratheses are numbers of differentially expressed genes identified. AGE-RAGE signaling pathway: advanced glycation endproducts-receptors for advanced glycation endproducts signaling pathway in diabetic complications; cGMP-PKG: cGMP-protein kinase G; CYP: cytochrome P450; ECM: extracellular matrix; ER: endoplasmic reticulum; MAPK: mitogen-activated protein kinase; PI3K-AKT: Phosphatidylinositol 3-kinase-protein kinase B; Th1 and Th2 cell differentiation: type 1 T helper and type 2 T helper cell differentiation.

The KEGG enrichment analysis was performed to identify pathways and biological functions associated with differentially expressed genes. The most up-regulated and down-regulated KEGG terms by the HFD at each time point are presented in Figures 6, 7. A greater number of significantly enriched KEGG pathways occurred at ZT 16 and ZT 20 of the dark phase (Figure 7). The up-regulated pathways included retinol metabolism, cholesterol metabolism, steroid and steroid hormone biosynthesis, bile acid synthesis and secretion, linoleic acid metabolism, glycine, serine, and threonine metabolism, tryptophan metabolism, protein processing in endoplasmic reticulum, pentose and glucuronate interconversions, glutathione metabolism, and ascorbate and aldarate metabolism. Pathway down-regulations occurred in focal adhesion, MAPK signaling pathway, lipolysis in adipocytes, Ras and Rap1 signaling pathways, P13-Akt signaling pathway, cell adhesion molecules, extracellular matrix-receptor interaction, and phospholipase D signaling pathway.

## Discussion

The rhythmicity analysis of differentially rhythmic transcripts showed that feeding pubertal mice the HFD for 3 weeks disturbed circadian rhythms in liver. More specifically, the HFD altered amplitude of rhythmic oscillations of *Clock*, *Bmal1*, *Rev-erbα*, *Per1*, *Per2*, and *Cry1*, elevated expression of *Clock*, *Per1*, and *Cry1*, and decreased expression of *Bmal1* and *Per2*. These results agree with a previous report that the HFD alters circadian gene expression in pubertal mammary glands (33).

Up-regulated *Per1* and down-regulated *Per2* expression by the HFD indicate that the HFD may alter genes of the *Per* family through different mechanisms. The *Per* gene expression is up-regulated by the *Clock/Bmal1* heterodimer and they, in turn, repress *Clock/Bmal1* expression. Previous research showed that the expression of *Per2* is not driven by peripheral oscillators in liver, but by the systematic feedback (34). Liver-specific *Per2* knockout leads to loss of food anticipatory activity and interferes with β-hydroxybutyrate production and its subsequent process in brain (35). β-Hydroxybutyrate is a byproduct of fat oxidation. However, β-hydroxybutyrate administration rescues food anticipatory activity in liver-knockout mice but not in whole body knockout mice, indicating a systemic regulation on *Per2* expression (35). Furthermore, a recent study showed differential expression of *Per2* between mice fed a high-sucrose diet and those fed a high-fat diet (36). Taken together, it suggests a role of dietary fat sensing in the expression of *Per* genes.

The present study showed that the expression of genes encoding lipid metabolism is in a diurnal pattern. The HFD decreased amplitude of *Acaca*, *Fasn*, and *Scd1* expression in liver. It suggests a decrease in *de novo* fatty acid synthesis, as a result of excessive fatty acids available from the diet. Acaca catalyzes the synthesis of malonyl CoA from acetyl-CoA, which is the first rate-limiting step in fatty acid synthesis. Fasn catalyzes biosynthesis of palmitate from condensation of acetyl CoA and malonyl CoA into long-chain saturated fatty acids (37). Scd1 catalyzes the desaturation of oleate and palmitoleate from stearoyl CoA and palmitoyl CoA, respectively. Furthermore, elevated expression of *Fads1* and *Fads2* occurred in HFD-fed mice, indicating an increase in synthesis of long-chain polyunsaturated fatty acids, which are essential components of plasma membranes (38, 39). Taken together, our findings showed that the HFD disturbed rhythmic expression of genes encoding lipid metabolism and the self-regulated controls of *de novo* fatty acid biosynthesis for lipogenesis in pubertal mice.

The protein-protein interaction network analysis clustered two groups of genes, those of circadian regulation and those of lipid metabolism, together by linking *Clock* and *Bmal1* in the circadian cluster to *Srebf1* in the lipid metabolism cluster. The *Srebf1* is a transcription factor that plays a key role in lipogenesis, facilitating the storage of fatty acids as triacylglycerols in liver by regulating expression of genes involved in lipid metabolism (40, 41). While the connection between the circadian genes and *Srebf1* must be considered preliminary because the relationship is based on text mining (22, 23) and because our results are transcriptional and not from direct measurement of protein expression, it does suggest that *Clock* and *Bmal1* or their dimers may regulate *Srebf1* during lipogenesis. Our findings support a previous study showing that the circadian clock modulates the promoter activity of *Srebf1* in hepatocyte nuclei from mice (42). Further investigations are warranted to understand the association between circadian regulation and *Srebf1*-mediated lipogenesis.

The HFD-induced metabolic disturbance mainly occurred in the dark phase of the day, specifically from ZT 16 to ZT 20. The KEGG enrichment analysis of differentially expressed genes showed up-regulations of signaling pathways related to retinol metabolism, fatty acid and lipid metabolism, amino acid metabolism and protein synthesis, and protection against oxidative damage. Down-regulations included pathways involved in diverse cellular processes, including cell proliferation, adhesion, migration, apoptosis, and cell survival.

In the present study, retinol metabolism is the leading up-regulated pathway in the dark phase by the HFD. Retinol is a precursor for biosynthesis of at least two critical metabolites, retinal and retinoic acid. Retinal is required for rhodopsin formation and vision (43). Retinoic acid functions as a ligand for nuclear retinoic acid receptors that regulates the expression of numerous genes for development and metabolism (44, 45). The retinoic acid metabolism occurs in two steps. The first is the oxidation of retinol to retinal by retinol dehydrogenase (46). The second is the oxidation of retinal to retinoic acid by retinal dehydrogenases (47). On a per calorie basis, the AIN93G diet and HFD provided equal amounts of nutrients including retinol to mice. We did not record food intake in this study because daily animal handling might disturb diurnal rhythms. However, we found in previous studies that energy intakes of post-pubertal mice fed the HFD are similar to that of mice fed the AIN93G diet (9, 30). Our findings suggest that the HFD may increase the production of retinoic acid that may be responsible, at least partly, for alterations in gene expression in the dark phase.

Up-regulations of steroid and steroid hormone biosynthesis and primary bile acid biosynthesis, as well as the up-regulated cholesterol metabolism, at ZT 16 and ZT 20 are evidence that the HFD accelerates cholesterol utilization in the dark phase. Cholesterol is the precursor for steroid and steroid hormone biosynthesis. The biosynthesis of bile acid involves modification of the cholesterol ring structure, conjugation, and removal of the side chain. Our findings of up-regulated bile acid biosynthesis and bile secretion support previous reports that HFD increases the total bile acid pool, which is likely a response to the increased dietary fat intake and availability of cholesterol from the diet (48). Furthermore, consistent with the up-regulated expression of *Fads1* and *Fads2*, the up-regulation of linoleic acid metabolism suggests an increase in biosynthesis of arachidonic acid, which is a constituent of phospholipids in plasma membranes (38, 39, 49). The up-regulated cholesterol metabolism in the dark phase suggests anabolic needs of mice in the active phase while the down-regulation of lipolysis may be a response to the increased availability of dietary fat.

The HFD up-regulated pathways of glycine, serine and threonine metabolism and tryptophan metabolism in pubertal liver. Metabolically, glycine and serine are interconvertible by serine hydroxymethyltransferase (50). Glycine is also an intermediate in threonine metabolism catabolized by threonine dehydrogenase to pyruvate (51–53). Tryptophan plays an important role in protein biosynthesis through the action of tryptophanyl-tRNA synthetase (54, 55). Furthermore, the HFD up-regulated protein processing in endoplasmic reticulum. It points to an increase in newly synthesized peptides to be glycosylated in endoplasmic reticulum before being transported to Golgi complex for further processing. These findings suggest that the HFD may increase non-essential amino acid and protein syntheses in pubertal liver, which may set the stage for further anabolic processes that will drive excess weight gain.

The observed up-regulation of the pentose and glucuronate interconversion pathway supports the notion of up-regulated non-essential amino acid and protein syntheses, as well as up-regulations of fatty acid metabolism. The pentose and glucuronate interconversion pathway plays an important role in many biosynthesis pathways, including transamination, deamination, lipogenesis, and gluconeogenesis (56).

The HFD up-regulated both glutathione metabolism and ascorbate and aldarate metabolism in the dark phase. Glutathione is an antioxidant that maintains cellular redox homeostasis (57). Ascorbate and aldarate metabolism is an important carbohydrate metabolic pathway that protects cells against oxidative damage from aerobic metabolism (58). Our findings indicate that the cellular protection against oxidative damage is rhythmic, which may be in a similar oscillating pattern to that of up-regulated metabolic pathways occurring during the active phase.

In the dark phase, the HFD down-regulated a number of signaling pathways that regulate diverse cellular processes, including cell differentiation, proliferation, cell adhesion, migration, apoptosis, and cell survival. These down-regulations included focal adhesion, MAPK signal pathway, Ras and Rap1 signaling pathways, the P13K-Akt signaling pathway, cell adhesion molecules, extracellular matrix-receptor interaction, and the phospholipase D signaling pathway. Taken together, these regulatory changes by the HFD that occurred particularly in the dark phase are evidence of the existence of coordinated mechanisms in rhythmic oscillations that temporally control the balance between metabolism and cellular maintenance.

In contrast to the dark phase, the HFD down-regulated signaling pathways related to lipid, protein, and energy metabolism at ZT 0. These pathways included fatty acid biosynthesis, steroid hormone biosynthesis, protein processing in endoplasmic reticulum and export, pyruvate metabolism, and carbon metabolism. It suggests that there is a transition in metabolism from the active dark phase to the resting light phase. In addition, there were no differences in these metabolic pathways between the AIN93G and HFD groups at the remaining time points of the light phase. These findings demonstrate further that the HFD-induced metabolic disturbance oscillates in a diurnal fashion with the most active in the dark phase of the day.

The observed circadian disturbance by the HFD with soybean oil as the source of dietary fat was similar to previous studies with lard as the dietary fat (14, 16, 59). We found previously that mice fed a soybean oil-based HFD consumed more diet in the light phase than in the dark phase (unpublished data), which is consistent with existing publications using lard-based HFD (14, 16, 59). These findings suggest that it is not the type of dietary fat but rather the temporal dysregulation of food intake pattern by the HFD that may disturb diurnal rhythms in mice. This is supported by findings that time-restricted feeding of an HFD to the dark phase restores metabolic rhythms in mice (10, 60).

A limitation of this study is that we did not analyze body composition because the magnetic resonance imaging analysis might alter diurnal rhythms in mice. Nevertheless, the observed rhythmic alterations in HFD-fed mice may be a direct effect of dietary fat, rather than an accumulation of body adiposity. This is supported by the findings of elevated hepatic triacylglycerols [soybean oil is a rich source of it (61)] and the null elevation of *Srebf1* in HFD-fed mice. This notion is further supported by the down-regulation of genes involved in *de novo* lipogenesis (e.g., *Acaca*, *Fasn*, and *Scd1*) in these mice.

In summary, feeding pubertal mice the HFD for 3 weeks altered hepatic metabolic rhythms with the greatest alteration in the dark phase. These alterations may disturb metabolic homeostasis during puberty and lead to metabolic abnormalities in these mice.

## Data availability statement

The original contributions presented in this study are publicly available. This data can be found here: https://www.ncbi.nlm.nih.gov/, GSE218932.

## Ethics statement

This study was reviewed and approved by the Institutional Animal Care and Use Committee of the Grand Forks Human Nutrition Research Center.

## Author contributions

LY and SS conceived and designed the study, performed experiments, collected data, and wrote the manuscript. DP and LJ performed statistical analyses. All authors contributed to the data interpretation and manuscript revisions and agreed the final version of the manuscript.

## Footnotes

1
https://fdc.nal.usda.gov/fdc-app.html#/food-details/2345748/nutrients

2
http://string-db.org
